# Association between hair dye use and human cancers: A systematic review

**DOI:** 10.1016/j.jdin.2025.10.009

**Published:** 2025-10-25

**Authors:** Rachel K. Greene, Jalal Maghfour, Cristina Nguyen, Gabrielle Baker, Natasha Atanaskova Mesinkovska

**Affiliations:** a Department of Dermatology, University of California Irvine, Irvine, California; b Department of Dermatology, Henry Ford Health, Detroit, Michigan; c Department of Medicine, University of California Los Angeles, David Geffen School of Medicine, Los Angeles, California; d School of Medicine, University of California, Irvine School of Medicine, Irvine, California

**Keywords:** bladder cancer, breast cancer, cancer, hair dyes, leukemia, neoplasm, non-Hodgkin’s lymphoma, paraphenylenediamine (PPD)

## Abstract

**Background:**

The global hair color market is valued over 23 billion dollars with over 2 billion in sales in the United States. Permanent hair dye accounts for approximately 80% of hair dye products on the market.

**Objective:**

To systematically review the association between hair dye use and cancer risk and identify vulnerable populations.

**Methods:**

A systematic search of PubMed and MEDLINE from January 1964 to March 2025 was conducted. Articles were reviewed independently by 3 assessors.

**Results:**

The review included 96 articles including 2 on both adults and children, and 5 on maternal exposure and pediatric cancer risk. Some studies suggested potential associations between hair dye use and cancer risk. Trends include increased risk of estrogen receptor + breast cancer among African American women and elevated bladder cancer in both genders. risk in frequent users. Individuals with slow acetylator N-acetyltransferase 2 genotypes or *CYP1A2* had elevated cancer risk with dye use. Maternal use during the first trimester significantly increased offspring risk of acute lymphoblastic leukemia further elevated by continued use during lactation.

**Limitations:**

Limitations include elements of study design, study populations, and confounders.

**Conclusion:**

There is evidence to suggest possible increased cancer risks for frequent, long-term hair dye use in specific populations.

**Capsule Summary**

• This review discusses the association between hair dye use and cancer risk, highlighting associations with dye type, frequency, and genetic factors.
• Dermatologists may wish to counsel patients on potential risks and safer practices, particularly for those considering or frequently using hair dye in susceptible populations.

## Introduction

The global hair color market is valued over 23 billion dollars with over 2 billion in sales in the United States. Asia dominated the market accounting for over 35% of global revenue in 2021. Hair dyes are classified by permanence: temporary, semipermanent, and permanent hair dye (PHD).^1^^,^^2^ PHDs account for approximately 80% of hair dye products on the market and contain coupler m-aminophenols that oxidizes and penetrates the hair cortex. Whereas, temporary and semipermanent do not oxidize therefore weakly adhere to the hair shaft.^2^ As a result, more consumers use PHD because it provides a more natural hair color than the other hair dyes.

The International Agency for Research on Cancer classifies occupational exposure to hair dyes as a probable carcinogen, though personal use remains unclassified.^1^ Certain chemicals found in hair dyes: 2,4-diaminoanisole, 4-amino-2-nitrophenol, and 4-chloro-o-phyenylenediamine are banned in Europe. Studies suggest certain chemicals including paraphenylenediamine (PPD) may be carcinogenic.^3^ This systematic review assesses the association between hair dye use including type, color, and cancer risk along with public health implications.

## Methods

**Fig 1:**
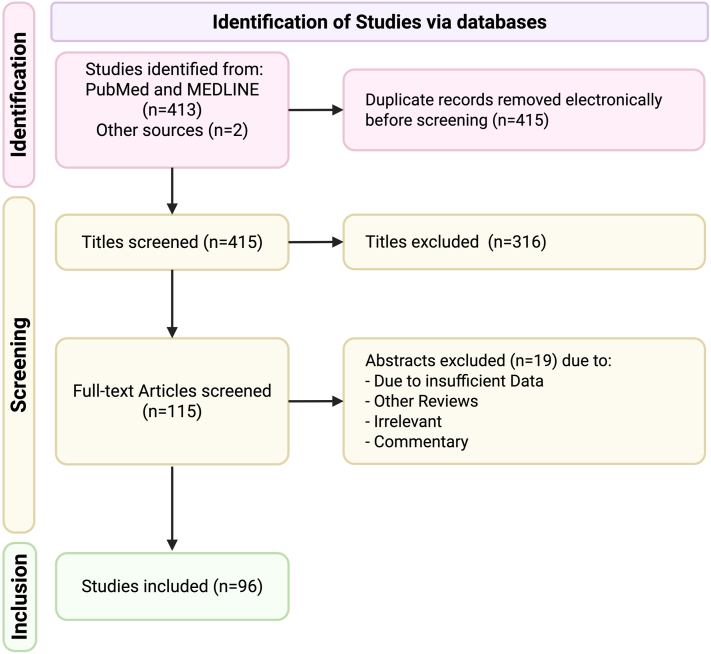
Preferred Reporting Items for Systematic Reviews and Meta-Analyses (PRISMA) flow diagram for the literature review of hair dyes and cancer

We conducted a systematic search using PubMed and MEDLINE with the Medical Subject Headings: hair dyes, hair colorants, hair color agents, cancer, and neoplasm. This systematic review protocol was registered at PROSPERO (CRD420251076719). We limited the search to English, human studies published between January 1964 and March 2025. Inclusion and exclusion criteria were specified before the literature search. To be included, the original articles needed to fulfill the following criteria: full-text, human studies investigating hair dye exposure as a risk factor for any type of cancer. We included clinically relevant randomized control, case control, prospective, and cohort studies. Exclusion criteria were non-English studies, secondary articles, letters to editors, commentaries, and experimental studies. Study design and outcome data were extracted and summarized in accompanying tables. We followed the Preferred Reporting Items for Systematic Reviews and Meta-Analyses guidelines (Fig 1), and graded studies according to the Oxford Centre for Evidence-Based Medicine 2011 Levels of Evidence.^4^ The Risk of Bias in Non-randomized Studies of Interventions tool was used to assess study risk, categorizing each as low, moderate, serious, critical, or no information (Table I, Table II, Table III, Table IV, Table V, Table VI).

**Table I:** Hair dye exposure and associated cancer risk

Cancer type	Level of evidence	Studies showing positive association *(n = number of patients)*	Studies showing no significant association *(n = number of patients)*	Controlled for major confounders
Breast^5^, ^6^, ^7^, ^8^, ^9^, ^10^, ^11^, ^12^, ^13^, ^14^, ^15^, ^16^, ^17^, ^18^, ^19^	2b-3b	9	7	Partial
Bladder^20^, ^21^, ^22^, ^23^, ^24^, ^25^, ^26^, ^27^, ^28^, ^29^, ^30^, ^31^, ^32^, ^33^, ^34^, ^35^, ^36^	2b-3b	7	11	Yes
Prostate^37^^,^^38^	3b	1	1	No
Gynecological^39^, ^40^, ^41^	2b-3b	3	0	Yes
Hematologic				
Leukemia^24^^,^^25^^,^^42^, ^43^, ^44^, ^45^, ^46^, ^47^, ^48^, ^49^, ^50^, ^51^, ^52^, ^53^, ^54^, ^55^, ^56^, ^57^	2b-3b	11	9	Partial
Myelodysplastic syndromes^58^^,^^59^	3b	2	0	No
Hodgkin lymphoma^24^^,^^25^^,^^42^^,^^49^^,^^50^^,^^60^^,^^61^	2b-3b	2	5	Yes
Non-Hodgkin lymphoma^10^^,^^24^^,^^25^^,^^42^^,^^48^, ^49^, ^50^, ^51^, ^52^^,^^54^^,^^56^^,^^61^, ^62^, ^63^, ^64^, ^65^, ^66^, ^67^, ^68^, ^69^, ^70^, ^71^, ^72^, ^73^	2b-3b	11	14	Yes
Multiple myeloma^24^^,^^25^^,^^27^^,^^49^^,^^50^^,^^60^^,^^61^^,^^74^, ^75^, ^76^	2b-3b	1	9	No
Brain neoplasms∗^,^^77^, ^78^, ^79^, ^80^, ^81^, ^82^, ^83^	3b	2	5	Partial
Melanoma^84^, ^85^, ^86^	2b-3b	1	2	Yes
Other†^,^^61^^,^^87^, ^88^, ^89^	3b	1	3	Partial

Summary of studies (*n* = 96) investigating the relationship between personal hair dye use and malignancy across organ systems.

Statistically significant positive association with cancer risk; No statistically significant association; Level of evidence based on Oxford Centre for Evidence-Based Medicine.

∗ Brain neoplasms include: glioma, glioblastoma multiforme, neuroblastoma, acoustic neuroma, meningioma.

† Other cancers include: salivary gland cancer, ovarian cancer, soft tissue sarcoma, testicular germ cell tumors; Controlled for major confounders (eg, smoking, occupational exposure): **Yes** = Most studies in this group controlled for these confounders, **Partial** = Some studies adjusted for them; others did not, **No** = Most or all studies did not adjust for major confounders.

**Table II:** The association between hair dyes and breast cancer

Author	Year of the report	Level of evidence	ROBINS-I rating∗	Study design	Study population	Findings
Cohort studies						
Henneckens et al^10^	1979	2b	Low	Retrospective cohort, 4 y of follow-up, 1972-1976	Cohort: 120,557; Cases: 270	Permanent hair dye use for (≥21 y) was associated with a significantly increased risk of breast cancer (RR = 1.38, *P* = .02).
Green et al^11^	1987	2b	Low	Prospective cohort, 6 y of follow-up, 1976-1982	Cohort: 118,404; Cases: 353	Permanent hair dyes RR = 1.1 (95% CI = 0.9-1.2) for ever use.
Eberle et al^5^	2020	3b	Moderate	Prospective cohort, average 8.3 y follow-up	Cohort: 46,709; Cases: 2794	**Permanent hair dye:** -Associated with a **9% increased breast cancer risk** overall (HR 1.09). -**Black women** showed a **45% increased risk** (HR 1.45); higher with frequent use (HR 1.60). -**White women** had a smaller increase (HR 1.07), significant with light-colored dyes (HR 1.12). **Chemical hair straighteners:** -Linked to an **18% higher breast cancer risk** overall (HR 1.18). **-Frequent use** (≥ every 5-8 weeks): **31% increased risk** (HR 1.31). Risk was similar across races. **Semipermanent dye** (nonprofessional use) -Associated with a **28% increased breast cancer risk** (HR 1.28).
Case-control studies						
Shore et al^9^	1967	3b	Moderate	Hospital-based, 1964-1976	Cases: 129; Controls: 193	Cumulative use associated with increased breast cancer risk, especially in women >50
Kinlen et al^12^	1977	3b	Moderate	Hospital-based, 1975-1976	Cases 191; Controls: 561	No association observed for permanent hair dyes (OR = 0.1); no trend with duration of use.
Stavraky et al^13^	1980	2b	Moderate	Hospital-based, 1976	Cases: London (*n* = 329), Toronto (*n* = 11,272) Controls: London (*n* = 314), Toronto (*n* = 470)	No significant association between use of permanent or semipermanent hair dyes and breast cancer overall; no dose–response relationship with usage frequency or color
Wynder et al^14^	1983	3b	Moderate	Hospital-based, 1979-1981	Cases: 401; Controls: 625	No association between hair dye use and breast cancer (OR = 1.02; 95% CI = 0.77-1.32); no significant dose-response with frequency, duration, type, or color, even after adjusting for confounders
Koenig et al^15^	1991	3b	Moderate	Hospital-based, 1977-1981	Cases: 398; Controls: 790	No significant increase in breast cancer risk with hair dye use (OR 0.8; 95% CI 0.6-1.1); no dose-response by frequency, duration, type, or color; same results for exposures >10 y prior
Nasca et al^16^	1992	3b	Low	Population-based, 1983-1990	Cases: 1617; Controls: 1617	Breast cancer was not found to be associated with ever use of hair dyes (OR = 1.04; 95% CI = 0.90-1.21).
Boice et al^16^	1995	3b	Low	Population-based, 1987-1996	Cases: 528; Controls: 2628	No significant association between breast cancer and ever-use of hair dyes (OR = 1.04; 95% CI: 0.90-1.21), nor with age at start/stop, duration, type, or lifetime applications
Cook et al^8^	1999	3b	Low	Population-based, 1983-1990	Cases: 844; Controls: 960	Higher risk (RR 1.9, 95% CI 1.4-2.5) of breast cancer seen in women using 2 or more coloring methods.
Petro-Nustas et al^17^	2002	3b	Moderate	Population-based, 1996	Cases: 100; Controls: 100	Higher odds of breast cancer associated with all hair dyes OR = 8.6 (95% CI = 3.3-22.3)
Zheng et al^18^	2002	3b	Moderate	Hospital-based, 1994-1997	Cases: 608; Controls: 609	No overall association with hair dyes OR = 0.8.
Llanos et al^6^	2007	3b	Low	Population-based, 2002-2008	Cases: 2280; Controls: 2005	-African American women: Dark hair dye use associated with 51% increased breast cancer risk (OR 1.51). -White women: Hair relaxer use linked to 74% increased risk (OR 1.74). -Combined dye + relaxer use: 2.4 × increased risk (OR 2.40). Dark dye also linked to ER + breast cancer in both groups.
Heikkinen et al^7^	2015	3b	Low	Population-based, 2000-2007	Cases: 6567; Controls: 21,598	Hair dye use was associated with a 23% higher breast cancer risk (OR 1.23, 95% CI 1.11-1.36), with stronger associations in women born before 1950 (OR 1.28) and a significant dose–response by cumulative use (*P* = .005)
Dianatinasab et al^19^	2018	3b	Moderate	Hospital-based, 2014-2016	Cases: 526; Controls: 526	Regular hair coloring associated with 93% increased breast cancer risk (OR 1.93; 95% CI 1.41-2.62)

*95% CI,* Confidence interval; *AA*, African American; *ER +*, estrogen receptor positive; *HR*, hazard ratio; *OR*, outcomes ratio; *ROBINS-I*, Risk of Bias in Non-randomized Studies of Interventions; *RR*, relative risk.

∗ Indicate the ROBINS-I domains or overall rating (low, moderate, serious, critical).

**Table III:** The association between hair dyes and bladder and prostate, and gynecological cancers

Author	Year of the report	Level of evidence	ROBINS-I rating∗	Study design	Study population	Findings
Bladder cancer						
Cohort studies						
Gubéran et al^90^	1985	3b	Moderate	Retrospective cohort, 28.9 y of follow-up, 1900-1982	Cohort: 20,336 (M), 21,903 (F); Cases: 703 (M), 677 (F)	Male hairdressers: no causal relationship between exposure to hair dyes and excess risk for bladder cancer.
Thun et al.,^25^ Altekruse et al^24^ Henley et al^26^	1999, 1994, 2001	2b	Moderate	Prospective cohort, 7 y of follow-up, 1982-1989	Cohort: 537,369; Cases: 336	Female: permanent hair dyes – RR = 0.65 and no trend with duration
Mendelsohn et al^27^	2009	2b	Moderate	Prospective cohort, 6-9-y follow-up	Cohort: 75,221; Cases: 32	Female: OR = 1.1; no association between hair dye use and bladder cancer RR = 1.14 (95% CI = 0.56-2.35) (adjusted for smoking)
Case-control studies						
Jain et al^28^	1977	3b	Low	Population-based, 1977	Cases: 107; Controls: 107	Permanent hair dye use: OR = 1.1 (95% CI = 0.41 to 3.03)
Neutel et al^29^	1978	3b	Moderate	Hospital-based	Cases: 50; Controls: 50	Female: all hair dyes OR = 0.9, no trend with frequency (not adjusted for smoking)
Howe et al^30^	1980	3b	Moderate	Population-based, 1974-1976	Cases: 632 (480 M, 152 F); Controls: 1:1	Female: all hair dyes OR = 0.7 Male: no controls exposed
Hartge et al^20^	1982	3b	Low	Population-based, 1977-1978	Cases: 2249 (M), 733 (M); Controls: 4282 (M), 1500 (F)	OR = 1.4 for dark color hair dyes (adjusted for smoking); all hair dyes OR = 0.9-1.1 (no trend with duration and frequency)
Sullivan et al^36^	1982	3b	Moderate	Population-based, 1977-1978	Cases: 82; Controls: 169	Increased incidence appeared in the number of times that hair dye was used per year for white female patients (*P* = .05) compared to matched controls
Ohno et al^31^	1985	3b	Moderate	Population-based	Cases: 293; Controls: 589	Use of all hair dyes was linked to a (OR = 1.5) higher risk, even after accounting for smoking
Gago Dominguez et al^23^	2003	3b	Low	Population-based, 1987-1996	Cases: 694 (M), 203 (F); Controls: 1:1	All hair dyes showed no association for males. Female: OR = 1.9 for permanent users (increasing trend with frequency and duration). OR = 2.9 (NAT2 slow phenotype), OR = 2.5 (CYPIS2 slow phenotype), and OR = 6.8 (non-NAT1∗10 genotype).
Andrew et al^21^	2004	3b	Low	Population-based, 1994-1998	Cases: 459; Controls: 665	Men: Inverse association with bladder cancer (OR = 0.5; 95% CI: 0.3-0.8) Women: Mildly elevated risk with permanent dye (OR = 1.5) and rinse dye (OR = 1.7)
Lin et al^32^	2006	3b	Low	Hospital-based, 1999-2001	Cases: 712; Controls: 712	Female: OR = 0.9 all hair dyes. Male: OR = 0.7 all hair dyes (adjusted for smoking)
Kogevinas et al^33^	2006	3b	Moderate	Hospital-based, 1998-2001	Cases: 128; Controls: 131	Female: all hair dyes OR = 0.8 (95% CI = 0.5-1.4) and permanent hair dyes OR = 0.8 (95% CI = 0.5-1.5) (adjusted for smoking)
Shakhssalim et al^34^	2010	3b	Low	Population-based, 2006	Cases: 692; Controls: 692	Hair dye use OR = 1.81 (95% CI = 1.08-3.06, *P* < .02)
Koutros et al^22^	2011	3b	Low	Population-based, 2001-2004	Cases: 1193, Controls: 1418	Increased risk of bladder cancer among users of permanent hair dyes with NAT2 slow acetylation phenotype (OR = 7.3, 95% CI = 1.6-32.6) compared to never users with NAT2 rapid/intermediate acetylation phenotype
Ros et al^35^	2012	3b	Low	Population-based, 2002	Cases: 1385; Controls: 4754	No clear association between hair dyes and cancer risk. Use of permanent hair dyes OR = 0.87 (95% CI = 0.65-1.18) and use of temporary hair dyes OR = 0.77 (95% CI = 0.58-1.02)
Prostate cancers						
Cohort studies						
Tai et al^37^	2016	3b	Moderate	Hospital-based, 2000-2008	Cases: 296; Controls: 296	Ever use of hair dyes for prostate cancer: OR = 2.15 (95% CI = 1.32-3.57); aged <60 y: OR = 2.13 (95% CI = 1.14-4.11); with hair dye use >10 y: OR = 1.97 (95% CI = 1.13-4.50), >6 times per year: OR = 1.97 (95% CI = 1.07-3.69), and hair dye use before 1980: OR = 0.62 (95% CI = 1.09-2.41)
Lim et al^38^	2022	3b	Low	Hospital-based, 1985-1988	Cases: 28,795	No significant association found between hair dye use and prostate cancer incidence (HR 0.99; 95% CI: 0.82-1.18) or mortality. No dose-response relationship with frequency or duration of use was observed.
Gynecological cancers						
Cohort studies						
White et al^40^	2021	2b	Low	Prospective cohort study, 2003-2009	Cases: 50,884	Frequent hair straightener use (≥4x/year) linked to increased ovarian cancer risk (HR 1.50; 95% CI: 1.02-2.20); no association found with hair dye or bleach use.
Chang et al^39^	2022	2b	Low	Prospective cohort study, 2003-2009	Cases: 33,947	Frequent use of chemical hair straighteners (>4x/year) associated with >2x increased risk of uterine cancer (HR 2.55; 95% CI: 1.46-4.45), with stronger risk among African American women.
Leung et al^41^	2023	3b	Moderate	Case–control study, 2011-2016	Cases: 491; Controls: 897	Increased ovarian cancer risk observed among hairdressers/beauticians (OR 1.34; 95% CI: 1.07-1.68), suggesting potential occupational exposure effects.

*95% CI,* Confidence interval; *F*, female; *HR*, hazard ratio; *M*, male; *NAT2*, N-Acetyltransferase 2 gene; *OR*, outcomes ratio; *ROBINS-I*, Risk of Bias in Non-randomized Studies of Interventions; *RR*, relative risk.

∗ Indicate the ROBINS-I domains or overall rating (low, moderate, serious, critical).

**Table IV:** The association between hair dyes and leukemia and myelodysplastic syndromes

Author	Year of the report	Level of evidence	ROBINS-I rating∗	Study design	Study population	Findings
Leukemia						
Cohort studies						
Grodstein et al^46^	1994	2b	Moderate	Prospective cohort, 8 y of follow-up, 1982-1990	Cohort: 99,067; Cases: 44	Ever use of hair dye for CLL: RR = 0.6 (95% CI = 0.3-1.5); ever use of hair dye for AML/CML/ALL: RR = 0.8 (95% CI = 0.3-1.9)
Thun et al.,^25^ Altekruse et al^24^	1994, 1999	2b	Moderate	Prospective cohort, 7 y of follow-up, 1982-1989; 12 y of follow-up, 1994	Cohort: 537,369; Cases: 718	Hair dye ever use: RR = 1.1; More than 20 y of use of brown color: RR = 1.5; More than 20 y of use: RR = 1.3
Mendelsohn et al^27^	2009	2b	Moderate	Prospective cohort, 5-9 y of follow-up, 1996-2005	Cohort: 75,221; Cases: 29	Hair dye ever use: RR = 0.7
Case control studies						
Cantor et al^51^	1988	3b	Moderate	Population-based, 1980-1983	Cases: 577; Controls: 1245	Ever use of hair dyes: OR = 1.8 (95% CI = 1.1-2.7)†
Zahm et al^49^	1992	3b	Moderate	Population-based, 1983-1986	Cases: 56; Controls: 1432	Ever use of hair dyes for CLL in F: OR = 1 (95% CI = 0.3-2.6); ever use of hair dyes for CLL in M: OR = 1 (95% CI = 0.2-3.8)
Mele et al^46^	1994	3b	Moderate	Hospital-based, 1986-1990	Cases: 252 (AML), 100 (ALL), 156 (CML); Controls: 1161	Female: AML use >10 y: OR = 1.6; ALL use >10 y: OR = 2.0; CML use >10 y: OR = 0.8 Male: dark color hair dye use for AML: OR = 1.6; dark color hair dye use for CML: OR = 2.1
Markovic-Denic et al^45^	1995	3b	Low	Population-based, 1989	Cases: 130; Controls: 130	Ever use of hair dyes for CLL: OR = 2 (95% CI = 0.96-3.26)
Miligi et al^50^	1999, 2005	3b	Moderate	Population-based, 1991-1993	Cases: 260; Controls: 828	Ever use of hair dyes: OR = 0.9; Ever use of dark permanent hair dyes: OR = 2.0; ever use of hair dyes for CLL: OR = 1.3 (95% CI = 0.8-2.2)
Bjork et al^55^	2001	3b	Moderate	Population-based, 1976-1993	Cases: 226; Controls: 251	Ever use of hair dyes for CML: OR = 0.4 (95% CI = 0.18-0.68)
Rausher et al^44^	2004	3b	Low	Population-based, 1986-1989	Cases: 769; Controls: 623	Ever use of hair dyes for ALL: OR = 1.3 (95% Ci = 0.99-1.8); light permanent hair dye: OR = 1.8 (95% CI = 1.1-3.1); dark permanent hair dye: OR = 1.6 (95% CI = 0.78-3.2)
Benavente et al^52^	2005	3b	Low	Population-based, 1998-2002	Cases: 574; Controls: 616	Permanent use of hair dye: OR = 2.3 (95% CI = 1.1-4.7)
De Sanjos et al^42^	2006	3b	Low	Population-based, 1998-2003	Cases: 2302; Controls: 2417	Ever use of hair dyes for CLL: OR = 1.43 (95% CI = 1.01-2.03)
Zhang et al^54^	2008	3b	Low	Pooled analysis	Cases: 4461; Controls: 5799	Hair dyes before 1980 for CLL: OR = 1.5 (95% CI = 1.1-2.0)
Wong et al^56^	2010	3b	Moderate	Hospital-based, 2003-2008	Cases: 694; Controls: 1298	Reduction in risk with NHL subtype (CLL): OR = 0.37 (95% CI = 0.18-0.76)
Rafieemehr et al^57^	2015	3b	Serious	Hospital-based, 2015-2018	Cases: 125; Controls: 130	Hair dye ever use for ALL: OR = 0.87 (95% CI = 0.32-2.37)
Parodi et al^48^	2016	3b	Moderate	Population-based, 2005-2009	Cases: 199; Controls: 279	Hair dye use >15 y for lymphoid malignancies: OR = 2.3 (95% CI = 1.0-4.9)
Karakosta et al^47^	2016	3b	Low	Population-based, 2009-2012	Cases: 138; Controls: 141	Hair dye use at home leading to CLL: OR = 0.99 (95% CI = 0.57-1.71)
Couto et al^43^	2018	3b	Low	Population-based, 1999-2007	Cases: 231; Controls: 419	ALL risk: Adjusted OR of 1.78 (95% CI: 1.13-2.81) for maternal exposure to hair dyes and straightening cosmetics (HDSC) during the first trimester of pregnancy. AML risk: Adjusted OR of 2.43 (95% CI: 1.13-5.22) for maternal exposure to HDSC during breastfeeding. MLL gene rearrangement: No association between maternal HDSC exposure and ALL or AML in children with MLL gene rearrangement.
Arshad et al^53^	2018	3b	Serious	Hospital-based, 2014	Cases: 25; Controls: 50	Ever use of hair dyes for leukemia: OR = 4.14 (95% CI = 1.28-4.95) in adults
Myelodysplastic syndromes						
Ido et al^59^	1996	3b	Serious	Hospital-based, 1992-1993	Cases: 116; Controls: 116	Ever use of hair dyes for females: OR = 2.5; Ever use of hair dyes for males: OR = 1.2
Nagata et al^58^	1999	3b	Moderate	Hospital-based, 1995-1996	Cases: 111; Controls: 830	Both genders forever use of any hair dyes: OR = 2.0†; ever use of hair dyes for females: OR = 2.9†
Hodgkin lymphoma						
Cohort studies						
Grodstein et al^60^	1994	2b	Moderate	Prospective cohort, 8 y of follow-up, 1982-1990	Cohort: 99,067; Cases: 24	RR = 0.9 for ever use of all hair dyes
Thun et al^25^ Altekruse et al^24^	1994	2b	Moderate	Prospective cohort, 7 y of follow-up, 1982-1989	Cohort: 537,369; Cases: 31	Use of black permanent hair dyes was associated with an increased risk: RR = 2.5 for 10-19 y of use, and RR = 2.1 for 20 or more years of use
Case-Control Studies						
Zahm et al^49^	1992	3b	Moderate	Population-based, 1983-1986	Cases: 70; Controls: 1432	Ever use of any hair dyes: Female: OR = 1.7 (95% CI = 0.7-4.0); Males: OR = 1.7 (95% CI = 0.4-6.3)
Miligi et al^50^	1999	3b	Moderate	Population-based, 1991-1993	Cases: 165; Controls: 828	OR = 0.7 (95% CI = 0.5-1.1) for ever use of any hair dyes or ever use of permanent hair dyes
Tavani et al^61^	2005	3b	Moderate	Hospital-based, 1985-1997	Cases: 158; Controls: 1295	OR = 1.14 (95% CI = 0/63-2.8) for ever use permanent hair dyes; OR = 0.7 (95% = 0.40-1.18) for ever use any hair dyes

*95% CI*, Confidence interval; *ALL*, acute lymphocytic leukemia; *AML*, acute myelocytic leukemia; *CLL*, chronic lymphocytic leukemia; *CML*, chronic myelocytic leukemia; *F*, female; *FL*, follicular lymphoma; *HDSC*, hair dyes and hair straightening cosmetics; *HR*, hazard ratio; *M*, male; *NHL*, non-Hodgkin lymphoma; *OR*, outcomes ratio; *ROBINS-I*, Risk of Bias in Non-randomized Studies of Interventions; *RR*, relative risk.

∗ Indicate the ROBINS-I domains or overall rating (low, moderate, serious, critical).

† 95% confidence interval excludes null value.

**Table V:** The association between hair dyes and Hodgkin and non-Hodgkin lymphomas

Author	Year of the report	Level of evidence	ROBINS-I rating∗	Study design	Study population	Findings
Hodgkin lymphoma (continued)						
Case-control studies						
De Sanjos et al^42^	2006	3b	Moderate	Population-based, 1998-2003	Cases: 2302; Controls: 2417	Ever use of hair dyes before 1980: OR = 1.75 (95% CI = 0.90-3.38)
Non-Hodgkin lymphoma						
Cohort studies						
Grodstein et al.	1994	2b	Moderate	Prospective cohort, 8 years of follow-up, 1982-1990	Cohort: 99,067; Cases: 144	Permanent hair dyes: RR = 1.1 (95% CI = 0.8-1.6) for NHL overall
Thun et al^25^	1994	2b	Moderate	Prospective cohort, 7 years of follow-up, 1982-1989	Cohort: 537,369; Cases: 763	Use of black hair dyes for 20 y: RR = 4.37 (95% CI = 1.3-15.2)
Altekruse et al^24^	1999	3b	Moderate	Prospective cohort, 12 years of follow-up, 1982-1994	Cohort: 547,586; Cases: 56	NHL: RR = 1.9 (95% CI = 1.0-1.3), hair due use (10-19 y): RR = 1.3 (95% CI = 1.0-1.6)
Case-control studies						
Cantor et al^51^	1988	3b	Moderate	Population-based, 1980-1983	Cases: 622; Controls: 1245	All hair dyes OR = 2.0 (95% CI = 1.3-3.0) for NHL, OR = 2.8 for follicular lymphoma. RR = 4.1 (95% CI = 0.9-20.9) for NHL for males who had used hair dyes at least once a month for a year
Zahm et al^49^	1992	3b	Moderate	Population-based, 1980-1986	Cases: 441; Controls: 1432	Female: permanent hair dyes OR = 1.7 for NHL overall and OR = 2.0 for follicular lymphoma. Male: no association
Holly et al^73^	1998	3b	Low	Population-based, 1991-1995	Cases: 1593; Controls: 2515	Female: no association; Male: semi-permanent hair dyes OR = 2.0 for NHL, OR 2.4 for DLBCL
Miligi et al^50^^,^^62^	1999, 2005	3b	Moderate	Population-based, 1991-1993	Cases: 611; Controls: 828	All hair dyes: OR = 1.0, permanent hair dyes: OR = 1.1 (95% CI = 0.9-1.4), follicular subtypes: OR = 1.3 (95% CI. = 0.8-2.0)
Schroeder et al^72^	2002	3b	Moderate	Population-based, 1980-1983	Cases: 182; Controls: 1245	All hair dyes: OR = 1.8 (95% CI = 0.9-3.7) for t(14;18) – positive NHL, OR = 2.1 (95% CI = 1.3-3.4) for t(14;18)-negative NHL
Zhang et al^10^^,^^54^	2004, 2009	3b	Low	Population-based, 1996-2000	Cases: 601; Controls: 717	Hair dye users before 1980: OR = 1.3 (95% CI = 1.0-1.8) for NHL; permanent hair dyes: OR = 1.9 (95% CI = 1.1-3.2) for follicular lymphoma; low grade B cell lymphoma: OR = 1.6 (95% CI = 1.0-2.5). Use of permanent hair dyes and dark shade increased risk of follicular lymphoma: OR = 1.3 (95% CI = 1.1-1.4)
Chiu et al^67^	2004	3b	Low	Population-based, 1980-1983	Cases: 807; Controls: 1926	All hair dyes OR = 1.4 for NHL, no data on NHL subtype
Tavani et al^61^	2005	3b	Moderate	Population-based, 1985-1997	Cases: 446; Controls: 1295	All hair dyes: OR = 1.0 for NHL; Ever use of permanent hair dye: OR = 1.25 (95% CI = 0.87-1.79); Semi permanent hair dyes: OR = 1.14 (95% CI = 0.77-1.67), no data on NHL subtype
Benavente et al^52^	2005	3b	Low	Hospital-based, 1998-2002	Cases: 574; Controls: 616	Ever use of hair dyes: OR = 1.2 (95% CI = 0.9-1.7); Permanent hair dye use: OR = 1.3 (95% CI = 0.9-1.9)
De Sanjos et al^42^	2006	3b	Low	Hospital- and population-based, 1998-2002	Cases: 2302; Controls: 2417	Hair dye use prior to 1980: OR = 1.4 (95% CI = 1.09-1.72), ever use of hair dyes: OR = 1.2 (95% CI = 1.0-1.41)
Chiu et al^66^	2007	3b	Moderate	Population-based, 1983-1986	Cases: 385; Controls: 1432	Hair dye use was not associated with follicular lymphoma or diffuse large B-cell lymphoma in either gender
Morton et al^68^	2007	3b	Low	Population-based, 1998-2000	Cases: 1321; Controls: 1057	Women who started using permanent, intense tone products (black, dark brown, dark blonde) before 1980: OR = 1.6 (95% CI = 0.9-2.7); Rapid/intermediate NAT2 phenotype: OR = 3.3 (95% CI = 1.3-8.6). Overall, no increased among Females and Males who started hair dye use after 1980.
Wong et al^56^	2010	3b	Moderate	Hospital-based, 2003-2008	Cases: 694; Controls: 1298	Ever use of hair dyes: OR = 0.9, FL: OR = 1.6
Chang et al^71^	2010	3b	Low	Population-based, 1981-1984	Cases: 622; Controls: 1245	Any hair dye use: OR = 1.3 (95% CI = 0.6-2.6) for t(14;18) positive NHL vs t(14;18) negative NHL: OR = 2.9 (95% CI = 1.6-5.0)
Sangrajrang et al^69^	2011	3b	Low	Population-based, 2007-2009	Cases: 390; Controls: 422	Ever use of hair dyes: OR = 1.1 (95% CI = 0.8-1.5), hair dye use before 1980: OR = 2.1 (95% CI = 1.0-4.1), risk of DLBCL with permanent hair dye use: OR = 1.6 (95% CI = 1.0-2.5)
Fan et al^65^	2012	3b	Low	Hospital-based, 2006-2010	Cases: 147; Controls: 294	Ever use of hair dye: OR = 0.9 (95% CI = 0.60-1.39); hair dye total frequency <20 y: OR = 0.95 (95% CI = 0.59-1.52); hair dye total frequency >20 y: OR = 0.79 (95% CI = 0.40-1.56)
Salem et al^64^	2014	3b	Moderate	Hospital-based, 2011-2012	Cases: 130; Controls: 130	*P* > .05 no significant association
Guo et al^70^	2014	3b	Low	Population-based, 1996-2000	Cases: 518; Controls: 597	Hair dye use before 1980: OR = 1.3 (95% CI = 1.0-1.8) – not observed for those who started using hair dyes in 1980 or later. Women who started using hair dyes before 1980 were noted to have increased risk of FL with various genotypes
Parodi et al^48^	2016	3b	Moderate	Population-based, 2005-2009	Cases: 199; Controls: 279	Hair dye use <15 y: OR = 1.4 (95% CI = 0.6-3.1), hair dye use >15 years: OR = 2.3 (95% CI = 1.0-4.9)
Kleinstern et al^63^	2017	3b	Low	Population-based, 2009-2014	Cases: 823; Controls: 808	Black hair dye use: OR = 1.7 (95% CI = 1.00-2.87); Israeli-Jews: OR = 1.13 (95% CI = 0.53-2.40); Palestinian Arabs: OR = 2.25 (95% CI = 1.03-4.91)

*95% CI,* Confidence interval; *DLBCL*, diffuse large b cell lymphoma; *F*, female; *M,* male; *NAT2*, N-Acetyltransferase 2 gene; *NHL,* non-Hodgkin lymphoma; *OR*, outcomes ratio; *ROBINS-I*, Risk of Bias in Non-randomized Studies of Interventions; *RR*, relative risk.

∗ Indicate the ROBINS-I domains or overall rating (low, moderate, serious, critical).

**Table VI:** The association between hair dyes and multiple myeloma and various neoplasms

Author	Year of the report	Level of evidence	ROBINS-I rating†	Study design	Study population	Findings
Multiple myeloma						
Cohort studies						
Grodstein et al^60^	1994	2b	Moderate	Prospective cohort, 8 y of follow-up, 1982-1990	Cohort: 99,067; Cases: 32	Hair dye use >20 y: RR = 0.4 (95% CI = 0.2-0.9)∗
Thun et al.,^25^ Altekruse et al^24^	1994, 1999	2b	Moderate	Prospective cohort, 7 y of follow-up, 1982-1989; 12 y of follow-up, 1994	Cohort: 537,369; Cases: 460	RR = 1.1 for hair dye ever use; RR = 3.1 for more than 20 y of use dark permanent hair dyes∗
Mendelsohn et al^27^	2009	2b	Moderate	Prospective cohort, 5-9 years of follow-up, 1996-2005	Cohort: 75,221; Cases: 18	Hair dye ever use: RR = 0.8
Case-control studies						
Brown et al^74^	1992	3b	Moderate	Population-based, 1981-1984	Cases: 173; Controls: 650	Hair dye use: OR = 1.9 (95% CI = 1.0-3.6)
Zahm et al^49^	1992	3b	Moderate	Population-based, 1983-1986	Cases: 72; Controls: 1432	Female: OR = 1.8 for ever using any hair dyes, OR = 2.8 for ever using permanent hair dyes. Male: OR = 1.8 for ever using any hair dyes
Herrinton et al^75^	1994	3b	Moderate	Population-based, 1977-1981	Cases: 689; Controls: 1681	Female ever use of any hair dyes: OR = 1.1 (95% CI = 0.80-1.30), Male forever use of any hair dyes: OR = 1.5 (95% CI = 0.75-2.90)
Miligi et al^50^	1999	3b	Moderate	Population-based, 1991-1993	Cases: 134; Controls: 828	Ever use of hair dyes: OR = 0.8 (95% CI = 0.50-1.20)
Tavani et al^61^	2005	3b	Moderate	Hospital-based, 1985-1997	Cases: 141; Controls: 1295	Ever use of any hair dyes: OR = 1.2 (OR = 0.70-1.97); ever use of permanent hair dyes: OR = 1.3 (95% CI = 0.74-2.19)
Koutros et al^76^	2009	3b	Moderate	Population-based, 1996-2000	Cases: 175; Controls: 679	Ever use of any hair dyes: OR = 0.8 (95% CI = 0.50-1.10)
Brain neoplasms (glioblastoma multiforme/glioma/neuroblastoma/acoustic neuroma/meningioma)						
Olshan et al^80^	1993	3b	Low	Population-based, 1984-1986	Cases: 200; Controls: 200	Maternal use of hair products during pregnancy for GBM: OR = 1.4 (95% CI = 0.7-2.9)
Bunin et al^79^	1994	3b	Moderate	Hospital- and population-based, 1986-1989	Cases: 155; Controls: 166	Maternal use of hair coloring products for astrocytoma: OR = 0.9 (95% CI = 0.3-1.6); maternal use of hair coloring products for PNET: OR = 1.1 (95% CI = 0.4-2.6)
Heineman et al^82^	2005	3b	Moderate	Population-based, 1988-1993	Cases: 112; Controls: 215	Ever use of hair dyes for GBM: OR = 2.4 (95% CI = 1.3-4.5; duration of exposure >21 y: OR = 4.9 (95% CI = 1.6-15.7)
Efird et al^77^	2005	3b	Low	Population-based, 1976-1994	Cases: 218; Controls: 2223	Maternal use of hair coloring agents in the month before or during pregnancy for CBT (OR = 1.0, 95% CI = 0.83-1.3), astroglial (OR = 1.1, 95% CI = 0.85-1.4), and PNET (OR = 1.0, 95% CI = 0.71-1.5)
McCall et al^78^	2005	3b	Moderate	Population-based, 1992-1994	Cases: 538; Controls: 504	Maternal hair dye use during the month before pregnancy and/or during pregnancy for neuroblastoma: OR = 1.6 (95% CI = 1.2-2.2); permanent hair dye use: OR = 1.4 (95% CI = 1.0-2.0); temporary hair use for neuroblastoma: OR = 2.0 (95% CI = 1.1-3.7)
Bluhm et al^83^	2007	3b	Moderate	Hospital-based, 1994-1998	Cases: 489 (GBM), 197 (meningioma), 96 (acoustic neuroma); Controls: 799	Use of permanent hair dyes for GBM: OR = 1.0 (95% CI = 0.6-1.6); long term use of brown permanent dye >20 y for GBM: OR = 3.8 (95% CI = 1.2-12.5). Ever use of hair dyes for meningioma: OR = 0.8 (95% CI = 0.5-1.4). Ever use of hair dyes for acoustic neuroma: OR = 0.9 (95% CI = 0.4-2.1)
Parodi et al^81^	2014	3b	Moderate	Population-based, 1970-1979	Cases: 104; Controls: 101	Hair dye use during pregnancy and neuroblastoma: OR = 3 (95% CI = 1.64-5.48)
Melanoma						
Cohort studies						
Vedel-Krogh et al^84^	2016	2b	Moderate	Prospective cohort, 27 y of follow-up, 1978-2005	Cohort: 7684 Cases: 1723	Malignant melanoma in F with versus without personal hair dye use: HR = 2.07 (95% CI = 1.25-3.42)
Case-control studies						
Osterlind et al^85^^,^^86^	1988,1990	3b	Moderate	Population-based	Cases: 474; Controls: 926	Ever use of any hair dyes: RR = 0.6 (95% CI = 0.5-0.9)
Other neoplasms						
Spitz et al^87^	1990	3b	Moderate	Hospital-based, 1985-1989	Cases: 64; Controls: 128	Females with any hair dye use for salivary gland cancer: OR = 4.1 (95% CI = 1.5-11.5); Males with any hair dye use for salivary gland cancer: OR = 0.7 (95% CI = 0.1-6/6)
Tzonou et al^88^	1993	3b	Moderate	Hospital-based, 1989-1991	Cases: 189; Controls: 200	Females with hair dye use (4 times per year) with ovarian cancer: RR = 1.74 (95% CI = 0.91-3.32); females with hair dye use (5 or more times per year) with ovarian cancer: RR = 2.16 (95% CI = 1.19-3.89)
Tavani et al^61^	2005	3b	Moderate	Hospital-based, 1985-1997	Cases: 221; Controls: 791	When compared to never use of any type of hair dyes for STS: OR = 0.73 (95% CI = 0.45-1.17)
Ghazarian et al^89^	2018	3b	Moderate	Population-based, 2002-2005	Cases: 526; Controls: 562	Maternal hair dye use (once per week versus less than once per week) for TGCT: OR = 0.8 (95% CI = 0.54-1.18)

*CBT,* Childhood brain tumors; *GBM,* glioblastoma multiforme; *HR*, hazard ratio; *PNET,* primitive neuro-ectodermal tumors; *ROBINS-I*, Risk of Bias in Non-randomized Studies of Interventions; *STS*, soft tissue sarcoma; *TGCT,* testicular germ cell tumors.

∗ 95% confidence interval excludes null value.

† Indicate the ROBINS-I domains or overall rating (low, moderate, serious, critical).

Three reviewers (J.M.,C.N.,G.B.) read abstracts to determine eligibility for inclusion in this systematic review. For each study, we recorded the study year, study design, level of evidence (LoE), study population and study findings.

## Results

### Study selection

**Fig 2:**
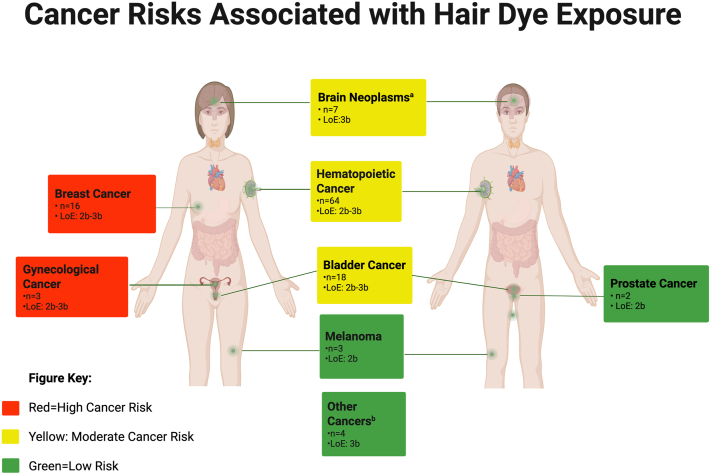
Heat map illustrating the association between hair dye exposure and cancer risk. Legend: Level of evidence (LoE) is based on the Oxford Centre for Evidence-Based Medicine guidelines. a) Brain tumors include glioma, glioblastoma multiforme, neuroblastoma, acoustic neuroma, and meningioma. b) Other cancers include salivary gland carcinoma, ovarian cancer, soft tissue sarcoma, and testicular germ cell tumors. *n*, Number of studies.

The literature search yielded 413 studies that met search criteria (Fig 1). After screening, 115 publications were reviewed for full-text eligibility with 2 more studies added based on bibliographic evaluation. Nineteen articles were excluded leaving 96 articles for inclusion. Thirteen articles are prospective cohort studies and 83 case-control studies. For the included studies, the LoE ranged from 2b to 3b (Fig 2).

Of the 96 studies, 87 evaluated adult patients, 2 included both adult and children, and 5 focused on maternal exposure and pediatric cancer risk. Analysis showed various cancers potentially associated with hair dye use: breast, bladder, lymphoma, hematopoietic, ovarian, childhood cancers, melanoma, prostate, salivary gland, glioblastoma, tenosynovial giant cell tumor, and soft tissue sarcoma (Table I).

#### Breast cancer

Most studies (n = 16, LoE:2b-3b) assessed the relationship between hair dye use and breast cancer (Table II). Overall, most studies report a direct association except for several cohort studies reporting associations in specific subgroups.

Cancer association with hair dye use varied by race particularly for PHD.^5^ Among African American (AA) women using hair dye every 5-8 weeks, breast cancer risk increased by 60% particularly in those using black colored PHD. In contrast, light colored-PHD was associated with significantly increased breast cancer among White women.^5^ Hair straightener use varies by race with AA women report using hair straighteners more frequently. Amongst this group, using hair straighteners every 5-8 week is associated with a 31% increased risk of breast cancer. Additionally, AA women have a 72% increased risk of estrogen receptor-positive disease using dark hair dye shades.^6^

Frequency and duration of hair dye use influences breast cancer risk. The cumulative frequency of semipermanent and PHD use is associated with increased risk of breast cancer, with odds ratios rising from 1.07 for 1-2 uses to 1.31 for 35-89 uses. Starting hair dye use at an earlier age, 20-29 years is associated with increased odds of breast cancer compared to those who began after age 40.^7^^,^^8^

The type and combination of hair coloring products are associated with increased breast cancer risk. Women using 2 or more types of hair dye products had a 3-fold increased risk if they accumulated 90 or more applications in their lifetime. Conversely, breast cancer risk is not strongly related among women who dyed their hair after bleaching or used frosting or rinses prior to dye application.^8^ Overall, breast cancer risk may be elevated among AA women, frequent and long-term users, and those using dark-colored or permanent dyes, particularly in relation to estrogen receptor-positive disease.

#### Bladder cancer

Some studies (*n* = 11; LoE:2b-3b) found no significant association between bladder cancer and hair dye use (Table III). However, the literature presents inconsistent findings with many studies limited by small sample sizes and population-based design.

A larger population-based study reports an increased risk of bladder cancer in both women and men with ever using black hair dye.^20^ Among women using PHD for over 31 years or over 282 applications have an increased risk of bladder cancer. On the other hand, men using any hair dye was inversely associated with any bladder cancer (odds ratio = 0.5; 95% CI = 0.3-0.8).^21^

Genetic susceptibility appears to modify the association between hair dye use and bladder cancer. Two studies report increased risk among individuals carrying slow acetylation N-acetyltransferase 2 (NAT2) genotypes or specific CYP1A2 genotypes. Exclusive PHD use was strongly associated with increased bladder cancer risk among individuals with the NAT2 slow acetylation genotype and those lacking the NAT1∗10 genotype.^22^ In contrast, no increased risk was observed among NAT2 fast acetylators.^22^ Similarly, PHD use was associated with a 2.5-fold increased bladder cancer risk among women with the slow *CYP1A2* genotype, an association not observed in the rapid genotype.^23^^,^^91^

#### Prostate cancer

Two studies (LoE = 3b) examine the association between hair dye use and prostate cancer (Table III). A study in Taiwan evaluated 296 prostate cancer cases with matched controls.^37^ Both studies did not distinguish between permanent, semipermanent, or temporary hair dyes. Exposure was assessed only as ever use versus never use. They report more than a twofold increased risk, particularly among men under 60 years of age who used hair dyes for over 10 years, applied them at least 6 times per year, and began use before 1980.^37^ Conversely, a Finnish cohort found no significant association between hair dye use and prostate cancer incidence or mortality.^38^

#### Gynecological cancer

Several studies (*n* = 3; LoE: 2b-3b) suggest an association between hair product use and gynecologic cancers (Table III).^26^ Women who used PHDs within the last 12 months had increased risk of nonserous ovarian tumors. Frequent use of hair straighteners was also reported within this cohort representing a potential confounding variable.^40^ Multiple studies included in this review identified a higher risk of ovarian cancer with frequent hair straightener use (≥4 times annually).^39^^,^^40^ Additionally, occupational exposure to hair chemicals may be relevant. Female hairdressers have a higher risk of ovarian cancer suggesting that cumulative exposure in a professional setting may contribute to gynecological cancer.^40^

#### Hematopoietic cancers

##### Leukemia

An association between hair dye use and leukemia risk has been reported in multiple studies (*n* = 11; LoE: 2b-3b) with variations in leukemia subtype and exposure patterns (Table IV).

PHD was associated with an increase in overall leukemia risk among long-term users (>20 years).^24^ Case-control studies reported stronger associations with chronic lymphocytic leukemia (CLL). Dark PHD increases CLL risk in females.^62^ Only CLL was significantly associated with the use of hair dyes and the risk of CLL increases with lifetime doses received.^52^ Furthermore, the risk of CLL increased among women using hair dyes before 1980.^24^^,^^52^ Hair dye use was associated with a 50% increased risk of developing acute lymphoblastic leukemia (ALL) with risks more than doubling among users with over 15 years of exposure.^44^

Conversely, some studies (*n* = 9; LoE:2b-3b) did not observe significant associations between hair dye use and certain leukemia subtypes including acute myeloid leukemia, and ALL highlighting inconsistencies in the current literature.^25^^,^^45^, ^46^, ^47^, ^48^, ^49^^,^^60^

### Myelodysplastic syndrome

Two case-control studies (LoE:2b) found an association between the risk of myelodysplastic syndrome and hair dye use(Table IV). Both studies did not distinguish between permanent, semipermanent, or temporary hair dyes. Exposure was assessed only as ever use versus never use. Risk was further elevated among individuals with both hair dye use and occupational exposure to organic solvents suggesting a synergistic effect.^58^ Neither study specified the color or type of dye used. The association between hair dye and myelodysplastic syndrome remained after adjusting for confounders like smoking and benzene exposure.^59^

#### Hodgkin’s lymphoma

The association between hair dye and risk of Hodgkin’s lymphoma (HL) (*n* = 5; LoE:2b-3b) largely reported no significant relationship (Tables IV and V). A 32-year prospective cohort study of women with biennial follow-up showed hair dye use was not associated with increased HL risk.^46^ Similarly, a case-control study reported elevated HL risk among PHD users with a comparable risk increase observed for those using dark dye shades odds ratio 3.4 vs 3.6, respectively.^67^ These findings suggest that while isolated studies have reported elevated risks with older formulations, the overall evidence does not support a consistent association between hair dye use and HL risk.^25^^,^^42^^,^^50^^,^^51^^,^^60^^,^^61^

#### Non-Hodgkin’s lymphoma

Some studies (*n* = 11; LoE:2b-3b) suggest a potential elevated risk of non-Hodgkin’s lymphoma (NHL) with hair dye use (Table V). Eight found no significant association while 4 studies noted increased risk among users before the 1980 formulations.^52^^,^^54^^,^^60^, ^61^, ^62^^,^^64^, ^65^, ^66^, ^67^, ^68^, ^69^^,^^92^

Black dye use and semi-PHD exposure were associated with elevated risk, particularly for diffuse large B-cell lymphoma.^9^^,^^48^^,^^54^^,^^92^ Using hair dye for at least 15 years is correlated with increased lymphoid malignancy risk among women.^48^ Follicular lymphoma, a subtype of NHL has been associated with the use of dark hair dyes. Increased risk was observed among individuals with exposure prior to 1980 and those with over 20 years of cumulative use.^54^^,^^68^^,^^69^^,^^92^

Four studies explored how genetic variation may modify the risk of NHL associated with hair dye use.^68^^,^^70^, ^71^, ^72^ Women with rapid or intermediate NAT2 genotype have significantly elevated NHL risk.^68^ Likewise, BRCA2 polymorphisms similarly increased follicular lymphoma risk.^70^ Several subtypes of NHL showed hair dye use associated with t(14;18) translocation and bcl-2+ lymphoma.^71^^,^^72^ Overall, NHL risk elevates with darker hair shades, pre-1980 formulations, long term use, and in genetically predisposed individuals.

### Multiple myeloma

Most studies (*n* = 9; LoE:2b-3b) found no association between hair dye use and multiple myeloma (MM) (Table VI). However, the largest cohort study found an increased MM risk with dark hair dye use (relative risk = 4.39; 95% CI: 1.1-18.3). Although the confidence intervals of the study are wide. Another study reported elevated MM risk with semi-PHD use in women and men.^25^ The literature presents inconsistent findings with many studies limited by small sample sizes.

### Cancers at other sites

Limited studies (*n* = 4; LoE:3b) have assessed PHD use and other cancers (Table VI). Using hair dyes for more than 15 years was linked to salivary gland carcinoma.^87^ Another study found a gene variant may be a key factor in renal cell carcinoma development when combined with hair dye use.^54^^,^^61^ Neither soft tissue sarcoma nor tenosynovial giant cell tumor noted a nonsignificant increased risk when using hair dye.^54^ A large cohort study observed ever usage of hair dyes reduced overall cancer mortality but elevated respiratory cancer risk among women with more than 20 years of use.^93^

### Hair dyes use in pregnant women and incidence of childhood cancer

Several studies (*n* = 5; LoE:2b-3b) explored maternal hair dye use and childhood cancers (Table VI). During the first trimester, hair dye use significantly increased ALL risk in offspring with even higher risks associated with use during breastfeeding.^43^ Two studies reported increased neuroblastoma risk: 1 showed 60% higher risk with use the month of conception and/or during pregnancy.^78^ Another study found a twofold increase of neuroblastoma associated with pregnant women using hair dye and work-related exposure during preconception.^81^ The 3 other studies found no significant links with Wilms tumor, astroglial tumors, or primitive neuroectodermal tumors.^77^^,^^79^^,^^80^

#### Brain tumors in adults

Two case-control studies (LoE:3b) found hair dye use associated with glioma and glioblastoma multiforme (Table VI).^79^^,^^80^ Risk of developing glioma and glioblastoma multiforme was associated with PHD exposure for 21 years or more.^82^ Use of dark-colored hair dye for over 20 years has been associated to an elevated risk of acoustic neuromas.^79^^,^^80^

##### Melanoma

A 37-year prospective study reports an increased melanoma risk with PHD.^82^ In contrast, 2 earlier case-control studies found no significant associations between melanoma and hair dye exposure (Table VI).^82^^,^^83^ Too few studies are available on melanoma to make a conclusion whether personal hair dye use is associated with melanoma.

## Discussion

Prior to 1980, hair dye formulations contained known carcinogens including aromatic amines used as dye intermediates. In response, manufacturers changed the ingredients of PHD to remove carcinogenic ingredients: 4-methoxy-m-phenylenediamine, 2,4-toluenediamine, and 2-nitro-p-phenylenediamine. Despite these changes, some current hair dyes contain PPD which may be carcinogenic.^94^, ^95^, ^96^ Additionally, DMDM hydantoin a formaldehyde releasing agent and known carcinogen remains in use for some current hair dye products.^97^ Occupational exposure also reflects this risk. Individuals working as a hairdresser for more than 10 years were nearly twice as likely to develop bladder cancer compared to those who never worked as a hair dresser.^98^ However, a recent study including Swedish hairdressers found no increased risk of bladder cancer in recent decades.^99^ This may suggest that modern hair dye formulations, which are free of aromatic amines may not confer the same level of risk. More research is needed to fully understand the long-term health effects of current hair dye formulations.

The relationship between hair dye use and cancer risk appears variable by cancer type (Table I). While the evidence for breast cancer, hematopoietic, and gynecologic cancers suggests elevated risks in specific groups. Certain populations may be disproportionately affected. Breast cancer risk was elevated among AA women and frequent users of dark PHDs. AA women demonstrated higher risks across breast and gynecological cancers, possibly reflecting combined exposures to hair dyes and chemical straighteners. Similarly, leukemia studies suggest subtype-specific risks with CLL risk rising with greater cumulative lifetime exposure. The greater number of studies on breast and bladder cancers may reflect early hypotheses from the 1970s linking aromatic amines and PPD in hair dye to endocrine and urothelial carcinogenesis.^1^ The relative paucity of studies on other malignancies represents the need to assess potential associations with less commonly studied cancers.

Genetic polymorphisms in detoxification enzymes such as NAT2 and CYP1A2 were shown to modify bladder cancer and NHL risk in susceptible individuals.^68^ This points to the interplay between genetic susceptibility and environmental exposure that may underlie cancer risk disparities and warrants further research.^68^ Among women rapid or intermediate NAT2 genotype have been associated with elevated NHL risk.^68^ While, BRCA2 polymorphisms have similarly been linked to an increased risk of follicular lymphoma.^70^ In bladder cancer, the use of PHDs was associated with increased risk among individuals with the *NAT2* slow acetylation genotype and those lacking the protective *NAT1∗10.* It may be beneficial to incorporate genetic screening for patients with significant or long-term hair dye exposure. This review is limited by heterogeneous endpoints, small sample sizes, recall bias, retrospective data and older dye formulations.

## Conclusion

Despite inconsistencies among the studies, the link between hair dye use and cancer risk merits further study. Additional studies are needed to evaluate the safety of current hair dye formulations and their long-term health effects.

## Conflicts of interest

None disclosed.
